# *Corynebacterium ulcerans* 0102 carries the gene encoding diphtheria toxin on a prophage different from the *C. diphtheriae* NCTC 13129 prophage

**DOI:** 10.1186/1471-2180-12-72

**Published:** 2012-05-14

**Authors:** Tsuyoshi Sekizuka, Akihiko Yamamoto, Takako Komiya, Tsuyoshi Kenri, Fumihiko Takeuchi, Keigo Shibayama, Makoto Kuroda, Masaaki Iwaki

**Affiliations:** 1 Laboratory of Bacterial Genomics, Pathogen Genomics Center, National Institute of Infectious Diseases, 1-23-1 Toyama, Shinjuku-ku, Tokyo, 162-8640, Japan; 2 Department of Bacteriology II, National Institute of Infectious Diseases, 4-7-1 Gakuen, Musashimurayama-shi, Tokyo, 208-0011, Japan; 3 Present address: Pharmaceutical and Medical Devices Agency, Tokyo, Japan

**Keywords:** Bacteriophage, Toxin gene, Horizontal gene transfer, Diphtheria, Zoonosis

## Abstract

**Background:**

*Corynebacterium ulcerans* can cause a diphtheria-like illness, especially when the bacterium is lysogenized with a *tox* gene-carrying bacteriophage that produces diphtheria toxin. Acquisition of toxigenicity upon phage lysogenization is a common feature of *C. ulcerans* and *C. diphtheriae*. However, because of a lack of *C. ulcerans* genome information, a detailed comparison of prophages has not been possible between these two clinically important and closely related bacterial species.

**Results:**

We determined the whole genome sequence of the toxigenic *C. ulcerans* 0102 isolated in Japan. The genomic sequence showed a striking similarity with that of *Corynebacterium pseudotuberculosis* and, to a lesser extent, with that of *C. diphtheriae*. The 0102 genome contained three distinct prophages. One of these, ΦCULC0102-I, was a *tox*-positive prophage containing genes in the same structural order as for *tox*-positive *C. diphtheriae* prophages. However, the primary structures of the individual genes involved in the phage machinery showed little homology between the two counterparts.

**Conclusion:**

Taken together, these results suggest that the *tox*-positive prophage in this strain of *C. ulcerans* has a distinct origin from that of *C. diphtheriae* NCTC 13129.

## Background

A diphtheria-like infectious disease caused by *Corynebacterium ulcerans* is increasing in clinical importance in developed countries and is now regarded as “diphtheria” in Europe [1,2]. Infection with *C. ulcerans* occurs in a wide range of hosts, including cats, dogs, pigs, cows, and whales [3-9]. The first clearly documented case of zoonotic transmission involved a dog, as reported by Lartigue et al. [5]. This is in contrast to the causative agent of classical diphtheria, *C. diphtheriae*, whose host species is thought to be limited to humans [10]. Nevertheless, the two species share a common feature: upon lysogenization of *tox*-encoding bacteriophages, they become toxigenic and are able to produce the potent diphtheria toxin [1,10]. This toxin is known to contribute to disease progression, occasionally leading to death. It is encoded by a single gene designated *tox*, situated inside prophages lysogenized in the bacterial genome of *C. diphtheriae*[11]. The prophages are capable of induction, by ultraviolet light or DNA-damaging agents such as mitomycin C, and yield β-, δ-, ω- and other functional bacteriophage particles [12]. Some types of bacteriophages can infect both *C. diphtheriae* and *C. ulcerans*[13-16]. Furthermore, the *C. ulcerans tox* gene is also encoded in a genome region surrounded by phage attachment (*att*) sites conserved between the two species [7,16]. The nucleotide sequences of *C. ulcerans tox* genes were published by Sing et al. They showed some diversity in the genetic sequence among *C. ulcerans* strains, in contrast to the highly conserved *C. diphtheriae tox* gene [17,18].

In 2003, the nucleotide sequence of the whole genome of *C. diphtheriae* strain NCTC13129 was reported [19]. The sequence information revealed some striking features of the bacterial genome, such as the presence of as many as 13 pathogenicity islands (PAIs) [19], uncommon among *C. diphtheriae* strains [20]. The presence of a *tox*-positive prophage flanked by the *att* regions was confirmed and supported the findings of previous reports [21]. Despite comparable clinical importance, the genomic sequence of toxigenic *C. ulcerans* has not yet been reported. In the present study, we determined the nucleotide sequence of the toxigenic *C. ulcerans* isolate 0102 genome, obtained in 2001 from the pharyngeal pseudomembrane of a 52-year-old woman presenting with a sore throat and fever. This was the first toxigenic *C. ulcerans* infection reported in Japan. This patient had been living with nearly 20 cats before the onset of illness [22]. Details of the bacteriological characteristics of the isolate have been described elsewhere [23]. Our analysis was especially directed towards the structure of the *tox*-positive prophage because of its unexpectedly novel structure.

## Results

### Genome sequence and genomic information for *C. ulcerans* 0102

To determine the complete genome sequence of *C. ulcerans* 0102, obtained short reads were assembled into five contigs by *de novo* assembly. Each gap was filled by direct PCR and sequencing. A circular chromosome sequence of *C. ulcerans* 0102 represents 2,579,188 bp, with a G + C content of 53.4% (Additional file 1) and corresponds to the predicted restriction fragment profiles obtained by PFGE analysis (Additional file 2). The chromosome possesses 2,349 coding sequences, 51 tRNA genes, and 4 *rrn* rRNA operons.

### Comparative genome analysis of three pathogenic *Corynebacterium* spp

**Figure 1:**
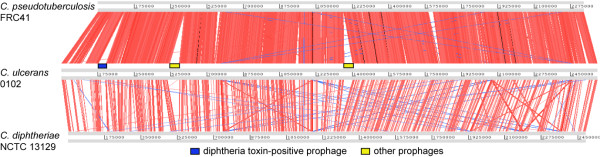
**Schematic genome comparison.***C. ulcerans* 0102 (middle) with *C. pseudotuberculosis* FRC41 (top) and *C. diphtheriae* NCTC 13129 (bottom) using a BLASTN homology search visualized by the ACT program. The red and blue bars between chromosomal DNA sequences represent individual nucleotide matches in the forward and reverse directions, respectively. BLASTN match scores less than 200 are not shown. A blue box and two yellow boxes represent a *tox*-positive prophage and other prophages on the chromosome of *C. ulcerans* 0102, respectively

Pair-wise sequence alignment revealed a highly conserved synteny among pathogenic *Corynebacterium* spp. (*C. pseudotuberculosis* FRC41, *C. ulcerans* 0102, and *C. diphtheriae* NCTC 13129; Figure 1). No significant genome rearrangements, such as inversion or transposition events, were observed among the three species, in accordance with previous findings [24]. The sequence similarity suggests that the chromosomes of *C. ulcerans* 0102 and *C. pseudotuberculosis* FRC41 are highly similar compared with that of *C. diphtheriae* NCTC 13129 (Figure 1). Once again, this is in accordance with previous findings in other *C. ulcerans* strains [24]. Similarly, a neighbor-joining phylogenetic tree, based on the partial sequence of *rpoB*, indicates that *C. ulcerans* 0102 is closely related with *C. pseudotuberculosis*, but clearly distinguishable from the *C. diphtheriae* clade (Additional file 3). Three prophages, ΦCULC0102-I, -II, -III, were identified in *C. ulcerans* 0102. One of the prophages, ΦCULC0102-I, carries *tox*, the gene encoding the diphtheria toxin (Figure 1).

### The *tox*-positive prophage of *C. ulcerans* 0102

**Figure 2:**
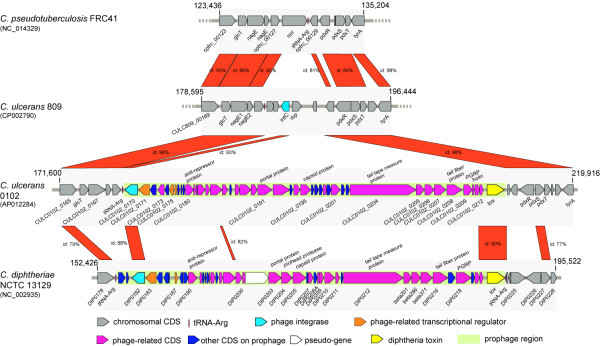
**Schematic representation and comparative analysis of*tox*-positive prophages and flanking regions.** The *tox*-positive prophage and flanking regions of *C. ulcerans* 0102 and *C. diphtheriae* NCTC13129 are shown. The corresponding region of *C. pseudotuberculosis* FRC41 and *C. ulcerans 809* is also shown. Boxes indicate individual coding regions with colors assigned to their functions. GenBank accession numbers are given in parentheses

The ΦCULC0102-I prophage of *C. ulcerans* 0102 is integrated into tRNA^Arg^ (CULC0102_t08) (Figure 2), suggesting that the integration site is identical to that in the *C. diphtheriae* NCTC 13129 corynephage. In contrast, the recently reported *C. ulcerans* 809 and *C. pseudotuberculosis* FRC41 genomes possess a phage-related integrase (*intC*) and a nitric oxide reductase (*nor*) gene, respectively, instead of a prophage (Figure 2). Putative attachment sequences were similar between both prophages carrying the *tox* genes (Additional file 4).

The two *tox-*positive prophages share the same structural features, with genes aligned in an ‘integrase - packaging - head - tail - lysis - toxin’ orientation (Figure 2). Pair-wise alignment of the prophages indicates a high similarity in the region encoding the putative integrase, the 3′-ends of CULC0102_0211 and CULC0102_0212, *tox*, and the attachment sites (Figure 2). The major phage machineries encoded in the internal phage region showed low similarity at the nucleotide and amino acid levels (less than 18%) between *C. ulcerans* 0102 and *C. diphtheriae* NCTC13129.

## Discussion

Whole-genome sequencing has revealed that the *C. ulcerans* 0102 genome is composed of 2,579,188 bp with a G + C content of 53.4%. These values are similar to those recently reported for *C. ulcerans* strains 809 (2,502,095 bp, 53.3% G + C) and BR-AD22 (2,606,374 bp, 53.4% G + C) [24]. *C. ulcerans* 0102 shares many common features with the two previously reported strains, including 12 virulence factors. Strain 0102 is distinctive with respect to the features of prophages integrated in its genome. It possesses a unique *tox*-positive prophage, ΦCULC0102-I, in its chromosome (Figure 1 and Additional file 1). In the same position of the recently reported *C. ulcerans* 809 genome exists a remnant phage-related integrase (*intC*) gene [24] (Figure 2). The *C. ulcerans* 0102 prophage differs from the corresponding prophage in *C. diphtheriae*. Although the integrase and *tox* gene sequences of ΦCULC0102-I showed high similarity to those of the corynephage encoding *tox* in *C. diphtheriae* NCTC 13129, the major phage machinery genes in ΦCULC0102-I are distinct from those in other corynephages in *C. diphtheriae* (Figure 2). This suggests that *C. ulcerans* 0102 did not immediately acquire the *C. diphtheriae tox*-positive corynephage.

**Figure 3:**
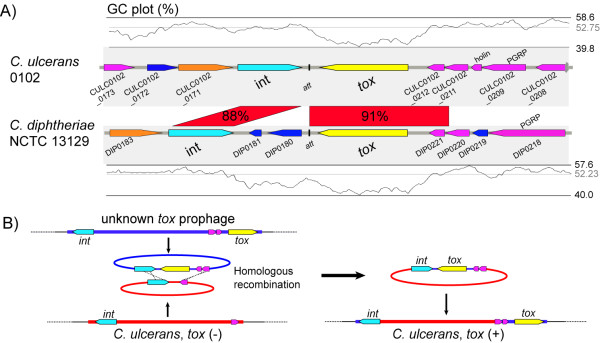
**Schema of the diphtheria toxin acquisition hypothesis.(A)** Pair-wise comparison of regions with high similarity between *C. ulcerans* and *C. diphtheriae*. These structures of putative phages are constructed by connecting attachment sites. The plots above and below represent the GC content calculated with a window size of 500 bp. **(B)** Schematic representation of how diphtheria toxin has been acquired in *C. ulcerans*

There are many possible explanations for the origins of these two prophages that are *tox*-positive but obviously different. One of the simplest explanations we can postulate is outlined in Figure 3. Generally, bacterial prophages are duplicated by excision from chromosomal DNA and subsequent concatenation at both ends of the *att* sites (Figure 3A). This duplication step indicates that two highly homologous regions, *int* and *tox,* could be in close proximity and adjacent to the *att* site concatenation. It could be speculated that homologous recombination between two prophages may facilitate the acquisition of the *tox* gene in *C. ulcerans* 0102 from an unknown *tox-*positive prophage (Figure 3B) [25]. Horizontal gene transfer is one of the major mechanisms of foreign gene acquisition by bacteria, as reviewed by Ochman et al. [26]. Liu et al. have demonstrated that horizontally transferred genes are often disabled and become pseudogenes. In these cases the genes are no longer beneficial to the recipients [27]. Non-toxigenic *C. diphtheriae* (CD450, CD119, CD448, and CD443 strains) carry *tox* pseudogenes that are relatively similar to the *tox* genes of *C. ulcerans* (Additional file 5), suggesting that horizontal gene transfer among *Corynebacterium* spp. might occur. Consistent with previous findings [7,17,18,28], tthe *tox* gene in *C. ulcerans* 0102 is not identical to that of *C. diphtheriae* (Additional file 5); phylogenetic analysis of *tox* showed greater heterogeneity among *C. ulcerans* isolates than that for *C. diphtheriae* isolates (Additional file 5).

The *C. diphtheriae tox* gene is highly conserved among temporally and geographically diverse strains [29], therefore greater variation in *tox* genes from *C. ulcerans* isolates suggests that this strain might have acquired the *tox* gene before *C. diphtheriae*.

In a recent report, whole genome sequence analysis of non-toxigenic *C. ulcerans* 809 and BR-AD22 [24], the β-corynephage-like truncated integrases (CULC809_00176 and CULC22_00173) are located adjacent to the tRNA^Arg^ gene, similar to ΦCULC0102-I in *C. ulcerans* 0102 and *C. diphtheriae.* The tRNA^Arg^ gene (CULC0102_t08) appears to be a ‘hotspot’ for the acquisition of ΦCULC0102-I-like prophages by homologous integrase.

The whole genome sequences of *C. ulcerans* 809 and BR-AD22 contain possible virulence factors, such as corynebacterial protease (CP40), phospholipase D (Pld), neuraminidase (NanH), venom serine protease (Vsp1), trypsin-like serine protease (TspA), Rpf interacting protein (RpfI), cell wall-associated hydrolase (CwlH), and five surface-anchored proteins (SpaB–F) [24]. The SpaA-type pilin, encoded by the *spaABCsrtA* gene cluster, is considered to play a crucial role in adhesion of *C. diphtheriae*[30]. The gene encoding the shaft protein of SpaA-type pilin (*spaA*) was absent in *C. ulcerans* 0102, a feature consistent with previous findings in *C. ulcerans* 809 and BR-AD2 [24]. As SpaB and SpaC proteins, which are assumed to be present in all three *C. ulcerans* strains, can contribute to host-cell adhesion in the absence of SpaA [30], this may imply a common mechanism of cell adhesion by *C. ulcerans*[24].

The *C. ulcerans* 809 strain was isolated from a patient with a rapid fatal pulmonary infection. The 809 strain-unique virulence factor (shiga toxin-like ribosome-binding protein, Rbp) is located adjacent to the truncated integrase (CULC809_00176) and corresponds to the integrase of ΦCULC0102-I. It appears that virulence factors have been acquired as a cassette gene in the ΦCULC0102-I-like prophage. It is intriguing to note that the 0102 strain does not carry the 809 strain-unique virulence factors (Rbp and the additional venom serine protease, Vsp2), but instead carries the *tox* gene on ΦCULC0102-I, which resulted in a diphtheria-like illness in a 52-year-old woman.

Isolates of *C. ulcerans* are generally obtained from a diverse range of animals, including humans. Isolation of a human pathogen *C. diphtheriae* from animals has been reported previously, although it is rare [31]. The *tox* gene might be frequently transmitted through common prophages with the aid of the highly homologous regions among *Corynebacterium* spp., including *C. diphtheriae* and *C. ulcerans* isolated from animal sources.

## Conclusions

Toxigenic *C. ulcerans* is an emerging pathogen that can be transmitted from animals to humans [5]. In the host organism, as well as in *C. diphtheriae*, the *tox* gene [18] is encoded by prophages. Through genome sequencing, we have identified a novel structure in a *tox*-positive *C. ulcerans* prophage with no significant sequence homology to those in *C. diphtheriae*. This suggests distinct origins of the prophages and thus may also explain the difference in the primary structures of their *tox* genes. The *tox*-positive bacteriophages may increase the dissemination risk of toxigenic *C. ulcerans* isolates, therefore, *C. ulcerans* isolates from both human and animal sources should be investigated further to determine the level of variation.

## Methods

This research was not carried out on humans. No experimental research on animals was carried out.

### Bacterial strain

The toxigenic *C. ulcerans* isolate 0102 was obtained in 2001 as a human clinical isolate [22,23].

### Preparation of genomic DNA

Genomic DNA was isolated by conventional methods, using phenol extraction and ethanol precipitation from heat-killed bacterial cells propagated in brain-heart infusion liquid medium.

### Short-read DNA sequencing using an Illumina Genome Analyzer IIx

DNA libraries of the ~600 bp insert length of *C. ulcerans* 0102 were prepared using a genomic DNA Sample Prep Kit (Illumina, San Diego, CA, USA). DNA clusters were generated on a slide using a Cluster Generation Kit (ver. 4) on an Illumina Cluster Station (Illumina), according to the manufacturer’s instructions. Sequencing runs for 80-mer short reads were performed using an Illumina Genome Analyzer IIx (GA IIx) and TruSeq SBS kit v5. Fluorescent images were analyzed using the Illumina base-calling pipeline RTA2.6/SCS2.8 to obtain FASTQ-formatted sequence data.

### De novo assembly of short DNA reads and gap-closing

The 80-mer reads were assembled (parameters k64, n51, c32.1373) using ABySS-pe v1.2.0 [32]. Predicted gaps were amplified with a specific PCR primer pair, followed by Sanger DNA sequencing using a BigDye Terminator v3.1 Cycle Sequencing Kit (Applied Biosystems, Foster City, CA, USA).

### Validation of the complete genome sequence using short-read mapping and pulsed-field gel electrophoresis (PFGE)

To validate the genome sequence, 40–mer short reads were re-aligned with the sequence using Maq software (ver. 0.7.1) and the *easyrun* Perl-command [33]. Read alignment was inspected using the MapView graphical alignment viewer [34]. PFGE analysis was performed to validate the predicted restriction fragment profiles from the complete genome sequence, according to De Zoysa et al. [35]. Bacterial cells were lysed with lysozyme and protease [36], embedded in plugs, digested with the restriction endonuclease *Sfi*I (New England Biolabs, Ipswitch, MA, USA) and electrophoresed in a CHEF DRII apparatus (Bio-Rad, Hercules, CA, USA) at 11°C with a pulse time of 5–20 s for the first 20 h and 1–5 s for the following 18 h.

### Annotation and pair-wise alignment analysis

Gene prediction from the complete sequence was performed using the NCBI Prokaryotic Genomes Automatic Annotation Pipeline (PGAAP; http://www.ncbi.nlm.nih.gov/genomes/static/pipeline.html). Several of the suggested errors were revised manually. Pseudogenes that were identified by PGAAP were checked using the read-mapping correction described above. Genomic information, such as nucleic acid variations and circular representation, was analyzed using IMC-GE software (Insilicobiology, Yokohama, Japan). A BLASTN homology search [37] was performed for the whole chromosome sequences of *C. pseudotuberculosis* FRC41 (accession no. NC_014329), *C. ulcerans* 0102, and *C. diphtheriae* NCTC 13129 (accession no. NC_002935). Aligned images of the homologous regions were visualized with the ACT program [38].

### Phylogenetic analysis

Phylogenetic analyses of all nucleotide sequences were conducted using the neighbor-joining method with 1,000-times bootstrapping in ClustalW2 [39]. FigTree ver. 1.3.1 (http://tree.bio.ed.ac.uk/software/figtree/) software was used to display the generated tree.

### Nucleotide sequence accession numbers

The complete chromosome sequence for the *C. ulcerans* 0102 strain has been deposited in the DNA Data Bank of Japan (DDBJ; accession no. AP012284).

## Competing interests

The authors declare that they have no competing interests.

## Authors’ contributions

TS and FT carried out the genome sequencing studies, participated in the sequence alignment and drafted the manuscript. TKo carried out maintenance, quality control and propagation of the bacterial strain for genome sequencing. AY and TKe participated in the design of the study. MT and KS conceived of and participated in coordination of the study, respectively. MK and MI coordinated the study, and drafted and finalized the manuscript. All authors read and approved the final manuscript.

## Supplementary Material

Additional file 1 Circular representation of the *C. ulcerans* 0102 genome. From the outside inward, the outer circle 1 indicates the size in base pairs (Mb). The red bars on Circle 2 show prophage region. Circles 3 and 4 show the positions of CDS transcribed in clockwise and anticlockwise directions, respectively. The dark blue bars on circle 5 indicate ribosomal DNA loci. Circle 6 shows a plot of G + C content (in a 20 kb window). Circle 7 shows a plot of GC skew ([G - C]/[G + C]; in a 20 kb window). Click here for file

Additional file 2 PFGE analysis of *C. ulcerans* 0102 with four restriction enzyme digestions. Click here for file

Additional file 3 Jukes-Cantor-derived phylogenetic tree based on the partial *rpoB* gene region among *Corynebacterium* isolates with 1,000-fold bootstrapping. Scale bar indicates number of substitutions per site. The number at each branch node represents the bootstrapping value. GenBank accession nos. given in parentheses. Click here for file

Additional file 4 Alignment of the nucleotide sequences of attachment site common regions among *C. ulcerans* 0102 and *C. diphtheriae* NCTC 13129. The red characters show regions annotated as tRNA^Arg^. Click here for file

Additional file 5 Phylogenetic tree based on the tox genes among toxgenic and nontoxigenic *Corynebacterium* spp. using the Neighbor-joining method with 1,000-fold bootstrapping. Scale bar indicates number of substitutions per site. The number at each branch node represents the bootstrapping value. GenBank accession nos. given in parentheses. Click here for file
