# Supplementary material for: Corynebacterium ulcerans 0102 carries the gene encoding diphtheria toxin on a prophage different from the C. diphtheriae NCTC 13129 prophage
Source: BMC Microbiol. 2012 May 14;12:72. doi: 10.1186/1471-2180-12-72 (PMC3406963; doi:10.1186/1471-2180-12-72)
Supplement: Additional file 1 — Circular representation of the C. ulcerans 0102 genome. From the outside inward, the outer circle 1 indicates the size in base pairs (Mb). The red bars on Circle 2 show prophage region. Circles 3 and 4 show the positions of CDS transcribed in clockwise and anticlockwise directions, respectively. The dark blue bars on circle 5 indicate ribosomal DNA loci. Circle 6 shows a plot of G + C content (in a 20 kb window). Circle 7 shows a plot of GC skew ([G - C]/[G + C]; in a 20 kb window). [file 1471-2180-12-72-S1.pdf]

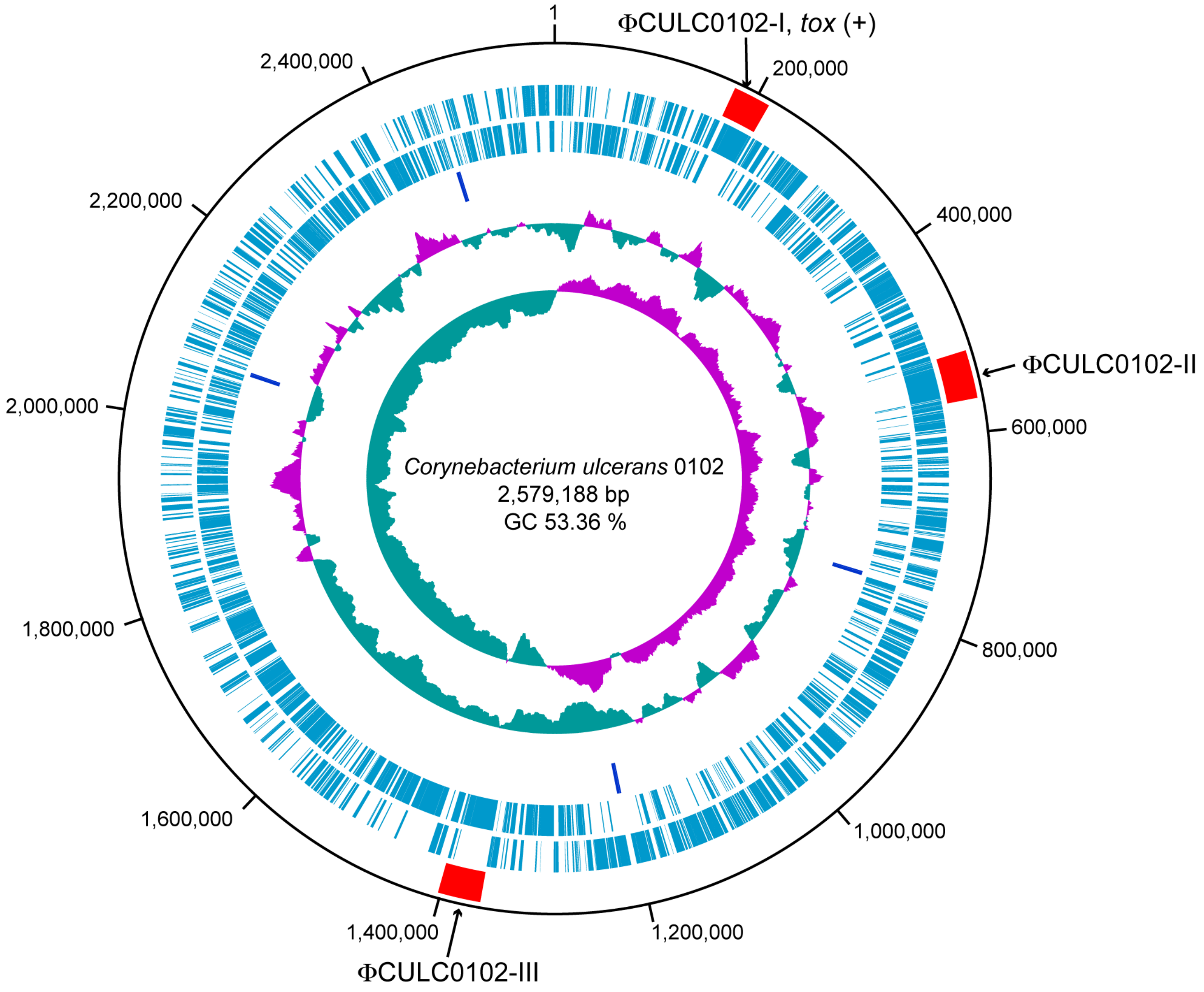

Additional file 1.

Circular representation of the *C. ulcerans* 0102 genome. From the outside inward, the outer circle 1 indicates the size in base pairs. The red bars on Circle 2 show prophage region. Circles 3 and 4 show the positions of CDS transcribed in clockwise and anticlockwise directions, respectively. The dark blue bars on circle 5 indicate ribosomal DNA loci. Circle 6 shows a plot of G+C content (in a 20 kb window). Circle 7 shows a plot of GC skew ([G - C]/[G + C]; in a 20 kb window).
