# Supplementary material for: Corynebacterium ulcerans 0102 carries the gene encoding diphtheria toxin on a prophage different from the C. diphtheriae NCTC 13129 prophage
Source: BMC Microbiol. 2012 May 14;12:72. doi: 10.1186/1471-2180-12-72 (PMC3406963; doi:10.1186/1471-2180-12-72)

Additional file2. PFGE analysis of *C. ulcerans* 0102 with four restriction enzyme digestions

*Fse*I

Cut site left	Cut site right	Predicted band (bp)	PFGE band (kb)	var
2,244,809	209,973	544,353	542	1.9
332,125	642,730	310,606	301	9.2
1,051,749	1,352,238	300,490	301	-1.0
2,029,151	2,244,808	215,658	210	5.5
1,712,475	1,914,277	201,803	199	3.2
783,724	926,924	143,201	143	0.4
1,446,744	1,587,659	140,916	143	-1.9
209,974	332,124	122,151	121	1.2
1,940,332	2,029,150	88,819	87	1.7
1,352,239	1,423,895	71,657	87	-15.4
1,645,220	1,712,474	67,255	66	1.3
642,731	695,862	53,132	48	5.4
695,863	747,922	52,060	48	4.3
1,004,330	1,051,748	47,419	48	-0.3
965,161	1,004,329	39,169	30	8.8
926,925	965,160	38,236	30	7.9
1,587,660	1,622,225	34,566	30	4.2
747,923	781,243	33,321	30	3.0
1,914,278	1,940,331	26,054	30	-4.3
1,622,226	1,645,219	22,994	30	-7.4
1,423,896	1,446,743	22,848	30	-7.5
781,244	783,723	2,480	14	-11.2
Total PFGE band (kb)			2,570	

*Pac*I

Cut site left	Cut site right	Predicted band (bp)	PFGE band (kb)	var
			849*	
1,766,813	2,309,870	543,058	530	13.5
2,478,184	292,943	393,948	382	12.2
1,202,085	1,547,945	345,861	333	12.6
483,288	740,495	257,208	250	7.2
1,547,946	1,766,812	218,867	214	4.6
292,944	483,287	190,344	185	5.8
2,309,871	2,478,183	168,313	161	7.8
1,050,454	1,202,084	151,631	143	8.8
906,865	1,050,453	143,589	138	5.7
806,629	906,864	100,236	89	11.0
740,496	806,628	66,133	60	6.4
Total PFGE band (kb)			2,484	

*Pme*I

Cut site left	Cut site right	Predicted band (bp)	PFGE band (kb)	var
2,338,841	226,150	466,498	465	1.4
461,575	798,453	336,879	332	5.1
2,019,447	2,242,252	222,806	222	0.8
241,624	461,574	219,951	222	-2.0
897,145	1,102,121	204,977	204	0.7
1,826,431	2,019,446	193,016	189	4.0
1,628,863	1,755,301	126,439	128	-1.7
1,505,909	1,622,828	116,920	114	2.9
1,154,244	1,270,707	116,464	114	2.5
2,242,253	2,338,840	96,588	95	2.1
1,412,822	1,505,908	93,087	95	-1.4
1,315,550	1,399,223	83,674	86	-2.5
1,755,302	1,826,430	71,129	68	3.1
798,454	866,714	68,261	68	0.3
1,270,708	1,315,549	44,842	42	3.0
1,102,122	1,146,541	44,420	42	2.5
866,715	897,144	30,430	42	-11.4
226,151	241,623	15,473	8	7.2
1,399,224	1,412,821	13,598	8	5.3
1,146,542	1,154,243	7,702	8	-0.6
1,622,829	1,628,862	6,034	8	-2.2
Total PFGE band (kb)			2,560	

*Swa*I

Cut site left	Cut site right	Predicted band (bp)	PFGE band (kb)	var
100,458	1,194,633	1,094,176	863	231.7
1,821,136	2,270,074	448,939	442	7.1
2,462,496	100,457	217,150	214	2.9
1,542,135	1,735,820	193,686	186	7.4
2,272,936	2,462,495	189,560	175	14.8
1,358,701	1,542,134	183,434	175	8.6
1,194,634	1,358,700	164,067	163	0.9
1,735,821	1,821,135	85,315	78	6.8
2,270,075	2,272,935	2,861	N.D.	-
Total PFGE band (kb)			2,296	

* Partial digestion fragment

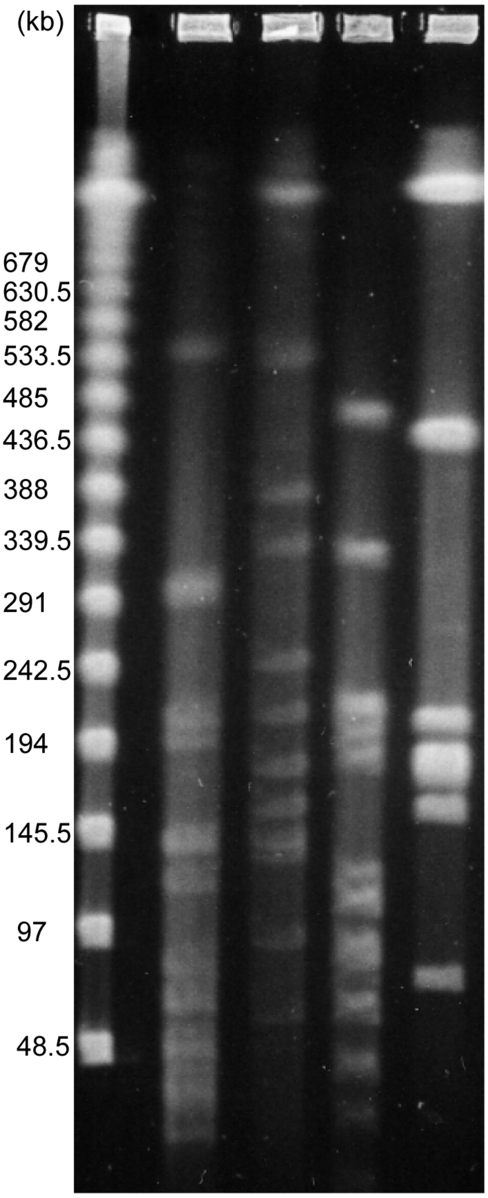

Supplement: Additional file 2 — PFGE analysis of C. ulcerans 0102 with four restriction enzyme digestions. [file 1471-2180-12-72-S2.pdf]
