# Supplementary material for: Corynebacterium ulcerans 0102 carries the gene encoding diphtheria toxin on a prophage different from the C. diphtheriae NCTC 13129 prophage
Source: BMC Microbiol. 2012 May 14;12:72. doi: 10.1186/1471-2180-12-72 (PMC3406963; doi:10.1186/1471-2180-12-72)
Supplement: Additional file 3 — Jukes-Cantor-derived phylogenetic tree based on the partial rpoB gene region among Corynebacterium isolates with 1,000-fold bootstrapping. Scale bar indicates number of substitutions per site. The number at each branch node represents the bootstrapping value. GenBank accession nos. given in parentheses. [file 1471-2180-12-72-S3.pdf]

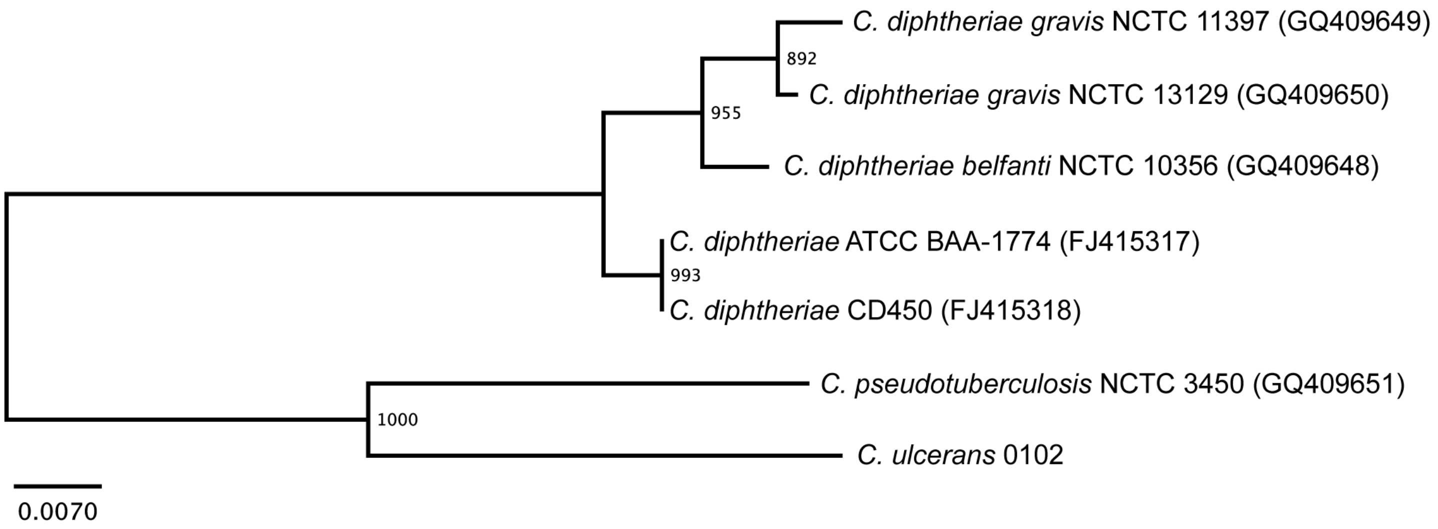

Additional file 3.
Jukes-Cantor-derived phylogenetic tree based on the partial *rpoB* gene region among *Corynebacterium* isolates with 1,000-fold bootstrapping. Scale bar indicates number of substitutions per site. The number at each branch node represents the bootstrapping value. GenBank accession nos. given in parentheses.
