# Supplementary material for: Corynebacterium ulcerans 0102 carries the gene encoding diphtheria toxin on a prophage different from the C. diphtheriae NCTC 13129 prophage
Source: BMC Microbiol. 2012 May 14;12:72. doi: 10.1186/1471-2180-12-72 (PMC3406963; doi:10.1186/1471-2180-12-72)
Supplement: Additional file 4 — Alignment of the nucleotide sequences of attachment site common regions among C. ulcerans 0102 and C. diphtheriae NCTC 13129. The red characters show regions annotated as tRNAArg. [file 1471-2180-12-72-S4.pdf]

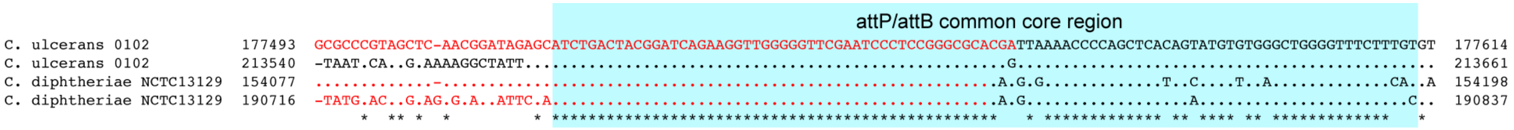

Additional file 4.
Alignment of the nucleotide sequences of attachment site common regions among *C. ulcerans* 0102 and *C. diphtheriae* NCTC 13129. The red characters show regions annotated as $tRNA^{Arg}$.
